# Physicians for Human Rights (UK)

**Published:** 1991-09

**Authors:** Christopher Burns-Cox


### PHYSICIANS FOR HUMAN RIGHTS (UK)

''No man committed a greater evil than to believe that because he could not do much he should do nothing''Edmund Burke.

Vital steps towards the reduction of civil rights abuses throughout the world must include their thorough investigation and maximum publicity.

Doctors and other health care workers have particular relevance regarding this. Some would say it was a duty.

Firstly, we should be proactive in general humanitarian matters as members of a profession whose aims include that of the reduction of human suffering.

Secondly, we have special privileges and skills easing access and enabling assessment of health status of individuals and populations.

Thirdly, we have a special relation with health care workers worldwide who suffer for their humanitarian beliefs and actions.

PHR (UK) was founded in 1989 and now has over 200 members including 45 Professors from Medical Schools throughout the UK. This year is has conducted two missions. The report on Kashmir 1991 confirms extensive civil rights abuses there and in particular details ways in which the health of the people, the health service and its staff have been harmed. The visit to Kuwait received widespread publicity. Other missions are planned as money becomes available. For this we urgently need more members, donations and offers to help.

Individual annual membership is £20 (£10 for students) and life membership £100 for those retired.

Please contact PHR (UK) c/o University of Forensic Medicine, The Royal Infirmary, Dundee DD1 9ND. (0384 200794).

Christopher Burns-Cox

